# Integrated cervical cancer screening in Mayuge District Uganda (ASPIRE Mayuge): a pragmatic sequential cluster randomized trial protocol

**DOI:** 10.1186/s12889-020-8216-9

**Published:** 2020-01-31

**Authors:** Carolyn Nakisige, Jessica Trawin, Sheona Mitchell-Foster, Beth A. Payne, Angeli Rawat, Nadia Mithani, Cathy Amuge, Heather Pedersen, Jackson Orem, Laurie Smith, Gina Ogilvie

**Affiliations:** 1 Uganda Cancer Institute, Kampala, Uganda; 2 Women’s Health Research Institute, Vancouver, Canada; 3 University of Northern British Columbia, Prince George, Canada; 4 University of British Columbia, Vancouver, Canada; 5 BC Centre for Disease Control, Vancouver, Canada; 6 BC Cancer, Vancouver, Canada

**Keywords:** Human papillomavirus, Cervical cancer, Human papillomavirus DNA tests, Visual inspection with acetic acid, Global health, Developing countries, Low resource setting, Self-collection, Cervical cancer screening

## Abstract

**Background:**

Cervical cancer is almost entirely preventable through vaccination and screening, yet remains one of the ‘gravest threats to women’s lives’ according to the World Health Organization. Specific high-risk subtypes of human papillomavirus (HR-HPV) are well-established as the primary cause of cervical cancer. Uganda has one of the highest cervical cancer incidence rates in the world (54.8 per 100,000) as a result of limited screening access and infrastructure. The integration of a self-collected cervical cancer screening program using HPV testing within existing community-based primary health care services could increase access to screening and reduce cervical cancer rates among Ugandan women.

**Methods:**

Using a pragmatic, sequential, cluster randomized trial design; we will compare the effectiveness of two cervical cancer screening models for self-collected HPV testing: 1) community health worker recruitment (door-to-door); and 2) community health meetings. In Mayuge district, Uganda, 31 villages are randomized to one of two treatment arms. Due to the nature of this trial, blinding is not possible. Women are eligible to participate if they have no previous history of hysterectomy or treatment for cervical cancer or pre-cancer and are aged 25–49 years old. All participants receive an integrated package of cervical cancer screening and education. Samples are tested for HPV using GeneXpert point of care testing. All women who test positive for HR-HPV types are referred to a designated health centre for follow-up inspection by Visual Inspection with Acetic acid (VIA) and treatment with thermal ablation. The primary outcome for the trial is the number of women who attend follow-up for VIA screening at a designated Health Centre after a positive HR-HPV test out of all women screened per arm. Secondary outcomes include: cervical cancer screening knowledge; patient-reported experience measures for self-collected cervical cancer screening; and HPV incidence.

**Discussion:**

Results from this study will inform the national scale-up of cervical cancer screening in Uganda, aligning with the World Health Organization’s target of achieving cervical cancer elimination through the pillar of increased HPV screening coverage.

**Trial registration:**

ISRCTN**,** ISRCTN12767014. Registered 14 May 2019, 10.1186/ISRCTN12767014; clinicaltrials.gov, NCT04000503; Registered 27 June 2019, https://clinicaltrials.gov/ct2/show/NCT04000503

**Protocol version:**

January 8, 2020, version 1.

## Background

Although cervical cancer is almost entirely preventable through vaccination and screening, it continues to be responsible for unnecessary deaths among women around the world. The World Health Organization (WHO) has called cervical cancer one of the ‘gravest threats to women’s lives’ [1] as it is the fourth most common cancer among women globally, with an estimated 570,000 new cases in 2018 [2]. The vast majority (over 85%) of cases occur in low- and middle-income countries (LMIC) with sub-Saharan Africa carrying the highest burden globally [2]. In Uganda, cervical cancer is the number one cause among women of both age-standardized cancer-related incidence (54.8 per 100,000) and cancer-related deaths (40.5 per 100,000) [3]. Challenges to provision of effective care in Uganda include competing health needs, misconceptions about screening, and poor prevention, screening, and treatment infrastructure, [4, 5] particularly in rural areas [4].

Specific high-risk subtypes of human papillomavirus (HR-HPV) are well-established as the cause of cervical cancer [6, 7]. Nearly all sexually active women are infected with HPV during their lifetime but most spontaneously clear the infection within 24 months [8, 9]. However, approximately 12% of these acute infections become persistent and can progress to precancerous lesions or invasive cervical cancer over decades, when not detected and/or treated early [10]. The extended natural history of HPV infection provides an opportunity for identifying effective screening programs to prevent cervical cancer.

The WHO recommends a screen-and-treat strategy to reduce incidence of cervical cancer. This strategy can include either screening with an HR-HPV test followed by visual inspection with acetic acid (VIA) and treatment with thermal ablation for women who test positive on both tests, or screening with an HR-HPV test and treatment for all HR-HPV positive cases [11]. HR-HPV testing provides the opportunity for specimens to be collected by either a clinician or the woman herself (self-collected), which may reduce individual and health system related barriers to screening, especially in low resource rural settings [11, 12]. Compared to no screening, the benefits of these strategies outweigh the harms; HR-HPV testing, however, produces a greater reduction in cancer and related mortality than VIA [11].

Self-collected HR-HPV testing is highly acceptable to women and care providers and has potential to be rapidly expanded through communities [12–15], particularly when offered door-to-door [12]. One of the identified barriers to scaling up self-collected CCS in rural LMIC settings is the shortage of health human resources. The WHO recommends a task shifting approach whereby tasks are transferred, when appropriate, from specialized workforce members to less specialized ones, such as community health workers (CHWs) [16]. CHWs are opportunely positioned to link their communities to health services which facilitates the decentralization of health care delivery to rural areas [16]. A systematic scoping review of CHWs roles in cervical cancer screening in LMIC indicates feasibility and acceptability of this approach, yet there is still a gap in the knowledge of how to implement this practice within community-based health services and its cost-effectiveness [17]. In order to optimize and ensure the elimination of cervical cancer, research conducted in LMIC that informs how to best implement self-collection in a low resource setting with high cervical cancer prevalence is needed.

## Methods

### Objectives

The primary objective of this study is to compare cervical cancer screening follow-up completion using two implementation approaches for self-collected HR-HPV testing in a rural, low-resource setting: 1) community health workers recruiting women door-to-door and 2) community health meetings. Secondary objectives of the trial are to compare cost-effectiveness of each cervical cancer screening model measured by the incremental cost-effectiveness ratio (ICER) and reduction in lifetime cervical cancer risk for each study arm and to conduct a process evaluation guided by the Reach, Effectiveness, Adoption, Implementation, Maintenance (RE-AIM) framework [18] focused on barriers and facilitators of implementation, implementation reach and fidelity for each model of cervical cancer screening used.

### Trial design

Advances in Screening and Prevention in Reproductive Cancers (ASPIRE) Mayuge is a pragmatic, sequential, two-arm cluster-randomized trial in Mayuge district, Eastern Uganda. The sequential nature of the study arms was chosen to prevent contamination between arms related to the enhanced community mobilization efforts included in arm 2. Ethics approval was granted from the University of British Columbia / Children’s and Women’s Health Centre of British Columbia Research Ethics Board (UBC C&W REB # H17–03332) and the Uganda Cancer Institute Research Ethics Committee (UCIREC REF-02-2018). All study participants have provided informed consent.

### Study setting

Mayuge district in Eastern Uganda has an estimated population of 480,000 residents divided into 13 sub-counties [19]. The majority of the population has access to health services through two level 4 health centres (HCIV) and five level 3 health centres (HCIII). Study activities take place in 31 villages found in two sub-counties in Mayuge district: 1) Buwaaya sub-country which includes the catchment population of Buwaiswa HCIII; 2) Mayuge town council (TC) which includes the catchment population of Mayuge TC HCIII. A full list of study sites can be obtained from the corresponding author. Health Centres included in this study have been supplied with the necessary equipment and materials to carry out their designated study activities. Study protocols were guided by the cervical cancer screening approaches laid out by the Uganda Ministry of Health including the purchase of a GeneXpert IV System for point of care testing. Research staff members carried out all training activities prior to recruitment.

The study involves task shifting self-collected CCS to CHWs, known locally as Village Health Teams (VHT). VHT are an existing element in Uganda’s community-based primary health care service model in rural areas and currently administer health education to communities through informal community health days. Topics for discussion at the community health days are determined through the demands of the local community or based on government defined priorities. For this trial, a total of 61 CHWs have completed a one-week training session guided by the WHO’s CHW training guidelines for CCS [20]. Training encompassed intervention procedures, recruitment strategies, effective communication, survey administration and proper documentation, epidemiology & pathology of cervical cancer, as well as screening & test results delivery. CHWs included in this study were recruited from the existing local health workforce and are a part of the local community in Mayuge.

Prior to recruitment, all laboratory staff at the Kigandalo HCIV were trained on the use of the GeneXpert IV System for HPV sample analysis including clinical utility, reagents, sample collection, kit storage and handling, preparing the cartridge, quality controls, and results analysis. Concurrently, a group of 7 nurses who provide VIA screening at three designated health centres (Kigandalo HCIV; Buwaiswa HCIII; Mayuge HCIII) were provided refresher training in cervical cancer screening, VIA, thermocoagulation, and quality monitoring.

### Participants and recruitment

**Fig. 1:**
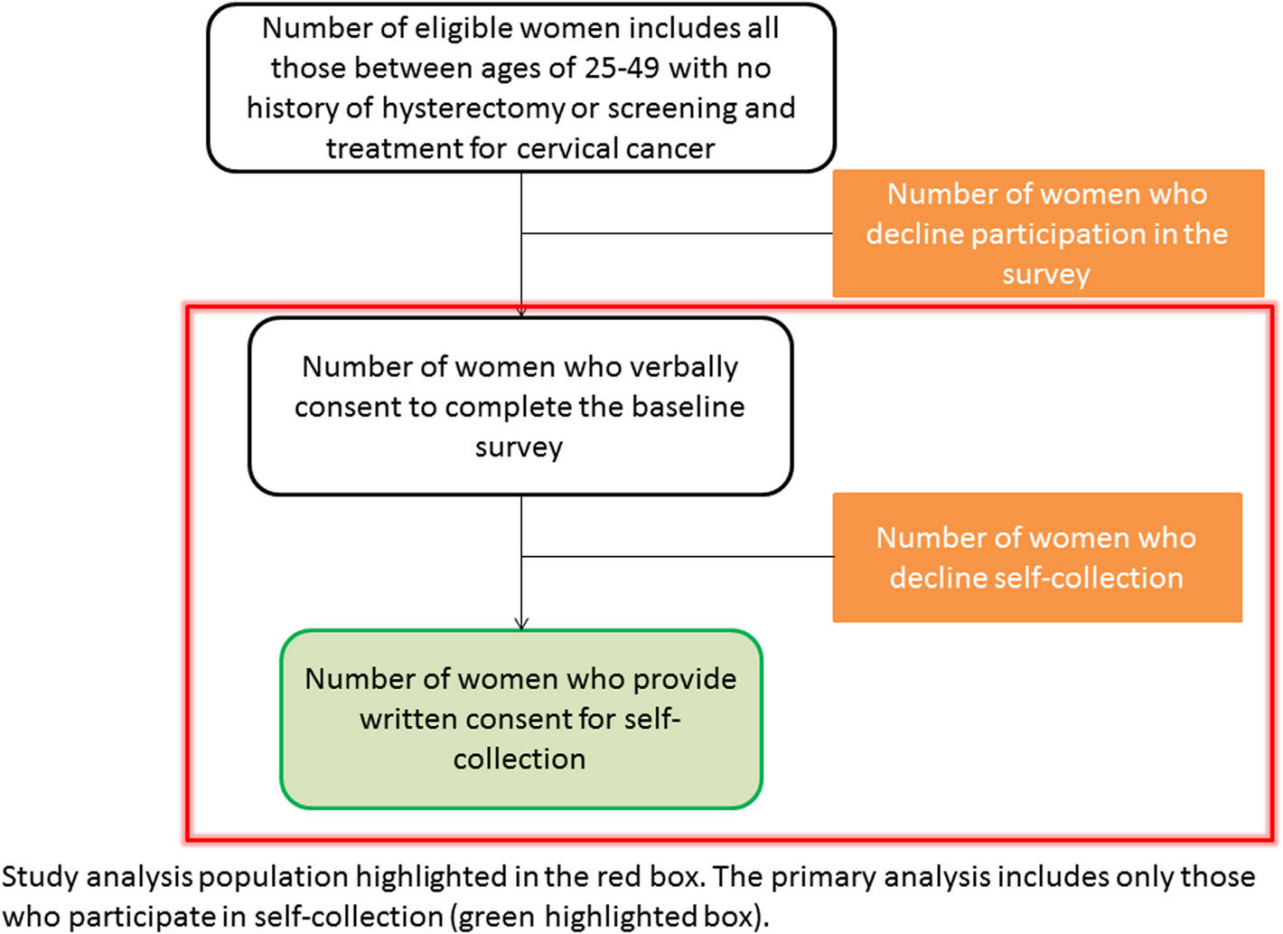
Recruitment flow

Women living in the Mayuge district are eligible to participate if they have no previous history of hysterectomy or treatment for cervical cancer or pre-cancer, are between the ages of 25 and 49 years, and have provided written informed consent. All women in the study villages who are approached and meet the inclusion criteria are invited to participate in a survey and self-collected HR-HPV testing. Women not meeting the inclusion criteria or those unable to provide informed consent are excluded. Any women who consent to the survey but decline self-collected CCS are referred to a HCIV in Kigandalo and are included in the study population but are not included in the primary analysis (Fig. 1). For arm 1, women will be recruited by a CHW going door-to-door. For arm 2, women will be recruited during a community health day meeting. Prior to the scheduled community health day meeting, CWHs will mobilize their own communities to attend the event by going door to door and by formally disclosing the planned activities to community leaders.

### Intervention

**Fig. 2:**
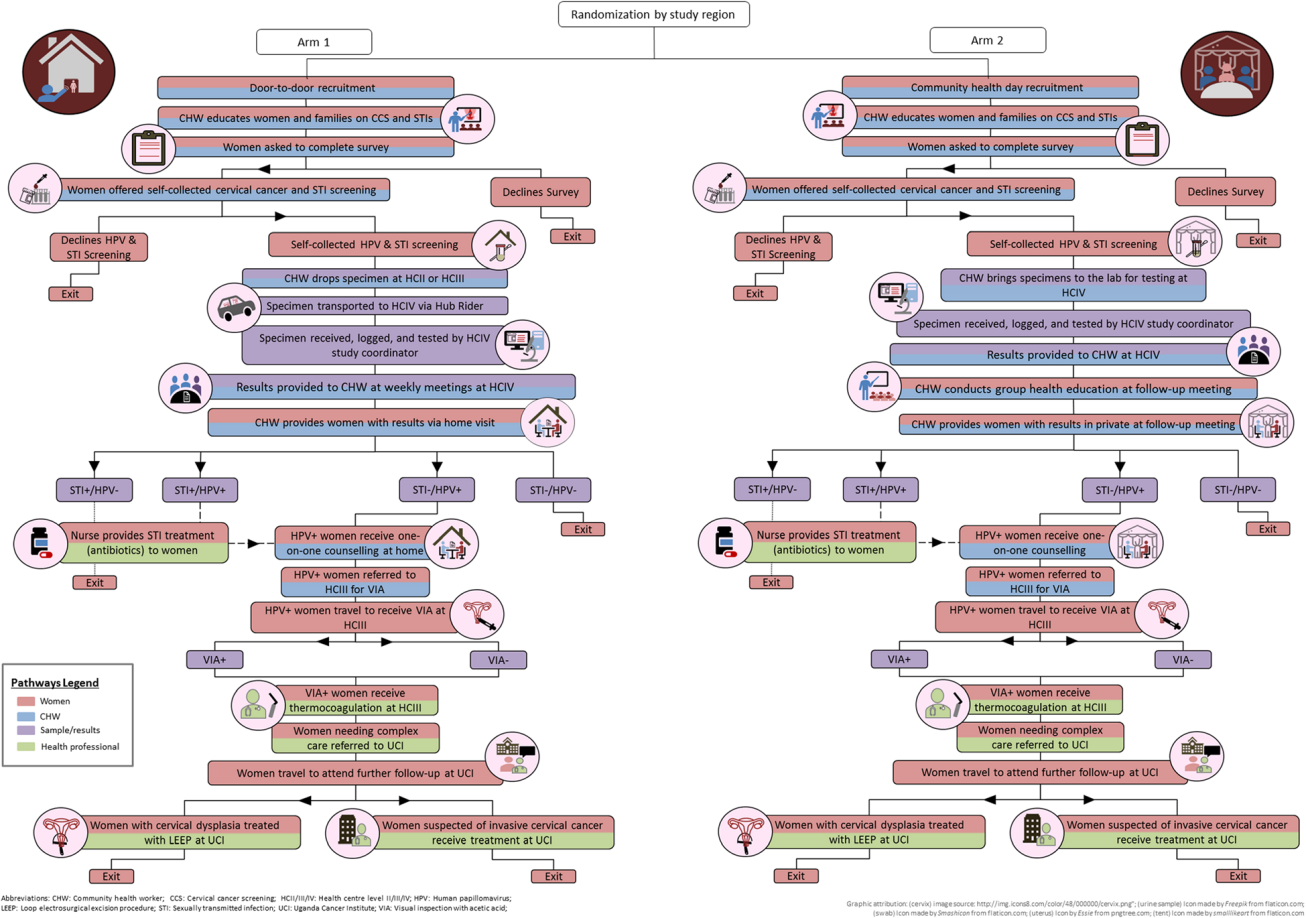
Pathways to Care Diagram. Legend describes who is involved in each step of the process

The ASPIRE Mayuge trial includes two sequential intervention arms. In both arms, participants are offered a package of self-collected cervical cancer screening and education that is integrated into community-based primary health care services, but interventions differ primarily by the model of delivery: door-to-door vs community health day. Participants who test positive for HR-HPV types follow the same pathway to care regardless of intervention allocation (see Fig. 2 for details). Standard VIA screening involves a practitioner applying 3–5% acetic acid to the cervix during a speculum exam. The practitioner then inspects and classifies lesions as positive, negative, or suspicious. Positive lesions are treated immediately with thermocoagulation. To align with the Ugandan Ministry of Health’s approach to CCS, in the ASPIRE trial all HR-HPV positive participants attending VIA follow-up are treated with thermocoagulation at an HCIII or HCIV, regardless of VIA result. Participants with suspicious lesions are referred to UCI for biopsy and Loop electrosurgical excision procedure (LEEP) or cancer treatment, if necessary. Transportation allowance is provided for participants to attend their follow-up visit. At the end of the intervention, a stakeholder meeting will be held to disseminate trial results.

To aid in the sustained uptake of screening behaviours, intervention activities incorporated a variety of theory-based health behaviour change techniques (BCT) described in Michie et al.’s Behaviour Change Techniques Taxonomy version 1(BCTTv1) [21]. The BCTTv1 characterizes 93 distinct BCTs clustered into 16 hierarchically structured categories to assist researchers in designing and describing behaviour change interventions [21]. (See Table 1).

**Table 1:** Behaviour Change Techniques

Component	Question	Method	Outcomes	Indicators	Behaviour Change Techniques
Program engagement (Education)	To what extent did the program shape participants’ knowledge and awareness of CCS?	CHW educates women and families on benefits and risks of cervical cancer screening.	Increased community awareness and knowledge of CCS	Knowledge and awareness scores compared between arms	3.2 Social support
					5.1 Information about health consequences
Program delivery (Screening)	How effective was the program at increasing CCS uptake among participants?	CHW instructs participants on self-collected CCS and offers them the opportunity for self-collected CCS.	Increased CCS among participants	Self-collected CCS uptake compared between arms	3.2 Social support
					4.1 Instruction on how to perform a behaviour
					6.1 Demonstration of the behaviour
					12.5 Adding objects to the environment
Program adherence (Follow-up and treatment)	How effective was the program at increasing CCS follow-up and treatment among participants?	CHW informs participants of their screening results. HR-HPV+ participants are referred for follow-up at HCII or HCIII and asked to set a date for follow-up/treatment.	Increased follow-up and treatment among HR-HPV+ participants	Follow-up/treatment attendance at each level of the pathway to care compared between arms	1.1 Goal setting (behaviour)
					1.4 Action planning
					2.6 Biofeedback
					3.2 Social support
					4.1 Instruction on how to perform a behaviour

### Arm1: door-to-door

In arm 1, participants receive one-to-one interaction with the CHW at their homes. A CHW goes door-to-door asking women to participate in a baseline survey, offers participants sexually transmitted infection (STI) and cervical cancer health education, and provides them with instructions on how to self-collect for CCS. The specimen is then dropped at a HCII or HCIII by the CHW and transported to a central laboratory at a local health centre for testing using GeneXpert point of care testing for HPV [22]. Samples are not banked and swabs are destroyed after testing. Following diagnostic testing, the CHW returns to the participants’ homes to provide them with their results. Participants who test positive for HR-HPV are invited to schedule their follow-up visit for VIA at an HCIII or HCIV.

### Arm 2: community health day

In arm 2, participants receive group interaction with a team of CHWs and other members of their community at a community health day meeting. Participants are asked to complete a baseline survey individually with a CHW. STI and cervical cancer health education and self-collected screening instructions are provided to participants in a group setting led by the CHW from their village. Participants choosing to complete self-collected CCS do so at a private location and a CHW drops the specimens at a HCIV at the end of the community health day. A follow-up community health day meeting is held to provide additional group education and CHWs deliver test results to participants in a one-on-one setting. Participants who test positive for HR-HPV are provided counselling and are invited to schedule their follow-up visit for VIA at an HCIII or HCIV.

### Subgroup interventions

To describe the association between HPV and other STI, a small sub-sample of 100 participants from each arm are provided the opportunity to self-collect for chlamydia and gonorrhea testing in addition to HR-HPV testing. For those who test positive for either of the STIs, treatment is dispensed by a nurse at the Health Centre for the patient and their partner. Treatment is provided according to National guidelines in Uganda.

### Study outcomes

Study outcomes were determined by applying RE-AIM framework [18] to intervention objectives. The primary outcome for this trial is measured at the individual level as positive or negative attendance for follow-up VIA screening (endline) at a designated Health Center after a positive HR-HPV test, among all participants screened per arm.

Secondary outcomes compared between arms include:

1. Cervical cancer screening knowledge measured as the count of correct responses to knowledge questions per individual within 6 months of recruitment.
2. Patient-reported experience measures for self-collected cervical cancer screening at intervention completion at the individual level within 6 months of recruitment.
3. HPV incidence at baseline as a sensitivity analysis to test our assumption of equal distribution of HR-HPV among the study population.

A summary of objectives, outcomes and analysis plans can be found in Table 2.

**Table 2:** ASPIRE Mayuge Evaluation Strategy

Research Objective	Research Question	RE-AIM Outcome	Outcomes	Data Analysis Approach
Primary Objective				
Self-collected cervical cancer screening effectiveness				
To compare the effectiveness of two self-collected CCS models at improving VIA follow-up: community health worker recruitment (door-to-door) versus community health day.	Which of the two self-collected CCS models is more effective at improving VIA follow-up among screened women: door-to-door screening or community health days?	Effectiveness (Individual level)	Primary Outcome: Follow-up attendance for VIA screening at a designated Health Center after a positive HR-HPV test out of all participants screened per arm	Quantitative analysis of clinical data: Mixed effect model with cluster as a random intercept and adjusted for all known confounders. Intention to treat and sensitivity analysis; Multivariate logistic regression
	What is the: prevalence of HR-HPV types; incidence of cervical cancer; association between HPV and STIs (gonorrhea and chlamydia); and risk difference in VIA follow-up between WLWHA vs non HIV?	n/a	HR-HPV prevalence; cervical cancer incidence; STI-HPV association; HIV-HPV association; Risk difference in VIA follow-up attendance between WLWHA vs. non HIV	Quantitative analysis of clinical and survey data: Descriptive statistics: prevalence, incidence; Bi-variate analysis: adjusted odds ratio, Risk difference (adjusted for cluster and other confounders)
	What is the effect of screening model on CCS knowledge retention and follow-up uptake? Are women aware of cervical cancer and how knowledgeable are they about CCS?	Effectiveness (Individual level)	Mean CCS knowledge scores; cervical cancer awareness;	Quantitative analysis of survey data: multi-level Poisson model;
	What are the motivators or inhibitors of CCS behaviour among women?	Effectiveness (Individual level)	Primary and secondary factors that motivate self-collected CCS; Primary and secondary factors that motivate VIA follow-up; Primary and secondary factors that inhibit VIA follow-up; Perceived social support from CHWs;	Quantitative analysis of survey data: descriptive statistics; Chi squared; multivariate logistic regression; Qualitative analysis of open-ended survey questions: deductive thematic analysis
Secondary Objectives				
Cost and feasibility				
To evaluate the cost and feasibility of a community-based CCS program in a low resource setting.	Which CCS model is more cost-effective?	Implementation (Setting level)	Cost-effectiveness of each CCS model (total provider, laboratory, transportation, equipment, training, and treatment costs per arm)	Quantitative analysis of facility survey data: ICER and reduction in CCS over lifetime; sensitivity analysis
	What are the costs associated with a CCS program?	Implementation (Setting level)	Monetary and time costs of CHWs and health care providers; training costs; laboratory costs; treatment costs; patient time costs;	Process evaluation: Narrative assessment/quantitative analysis of study logs: Descriptive statistics - univariate analysis (frequencies)
Best Practices for integrated community care				
To identify the barriers and facilitators of implementation, implementation reach, and fidelity for each model of CCS.	What are patients’ preferences for integrated service delivery? (barriers/facilitators of implementation)	n/a	Patients’ preferences for integrated service delivery	Quantitative analysis of survey data: Chi squared; multivariate logistic regression
	What is the acceptability of a community-based CCS program among participants? (barriers/facilitators of implementation)	Effectiveness (Individual level)	Patient-reported experiences with a community-based CCS program	Quantitative analysis of survey data: Descriptive statistics with time from sample collection to patient experiences survey as offset; Qualitative analysis of open-ended survey questions: deductive thematic analysis
	What were the CCS program inputs? (Implementation reach)	Implementation (Setting level)	Total program inputs (financial, human, administrative, equipment resources)	Process evaluation. Narrative assessment/quantitative analysis of study logs: Descriptive statistics - univariate analysis (frequencies)
	What was the reach of the program? (Implementation reach)	Reach (Individual level)	Participation at each level of the pathway to care; stakeholder engagement; Survey participation; sociodemographic characteristics of participants	Process evaluation. Quantitative analysis of survey, study log, and clinical data: Descriptive statistics - univariate analysis (proportion, frequency, mean); Chi squared; T-test;
	How many participants were lost to follow-up? (fidelity)	Maintenance (Individual level)	Attrition at each level of the pathway to care	Process evaluation. Per-protocol analysis; Quantitative analysis of survey, study log, and clinical data: Descriptive statistics - univariate analysis (frequency); sociodemographic characteristics of those lost to follow-up
	Was the CCS program implemented as intended? (fidelity)	Implementation (Setting level)	Planned vs actual intervention components (e.g. number of training sessions, number of specimens transported and tested, etc.)	Process evaluation. Quantitative analysis of study logs and clinical data: Descriptive statistics - univariate analysis (mean, frequency)
	How successful was VIA training and quality monitoring during the trial? (fidelity)	Implementation (Setting level)	Detection rates of CIN2+ lesions over time; adverse and serious adverse events; Themes related to health care workers experiences;	Quantitative analysis of clinical data: descriptive statistics - univariate analysis (frequency); qualitative analysis; qualitative analysis of FGD data: deductive thematic analysis
	How acceptable and feasible is the HPV screen and treat approach to women and health workers? (barriers/facilitators of implementation)	Implementation (Individual level)	Treatment rate vs. VIA + rate; patient reported experience measures from treatment; themes related to health care workers experiences	Quantitative analysis of clinical data: descriptive statistics - univariate analysis (frequency); qualitative analysis; qualitative analysis of FGD data: deductive thematic analysis
	How representative were the included villages (clusters)?	Adoption (Setting level)	Participation, exclusion, and representativeness of included villages in Mayuge district	Literature review of Uganda National Planning Authority data
	What modifications were made to the study’s original CCS program to meet the National program’s interests?	Maintenance (Setting level)	Modifications to original CCS program plans of the study to align with Uganda’s national CCS program interests	Narrative assessment of study’s program planning activities and National program interests
Other Objectives				
Men’s role in cervical cancer screening				
To understand the role that men play in CCS	How knowledgeable are men about HPV and cervical cancer?	n/a	HPV and cervical cancer knowledge	Qualitative analysis of survey data: Descriptive statistics - univariate analysis (mean)
	What are men’s attitudes/perceptions of cervical cancer and screening?	n/a	Attitudes and perceptions of cervical cancer and CCS	Qualitative analysis of survey data: Descriptive statistics - univariate analysis (frequency)
	What factors impact men’s supportiveness towards their partner seeking cervical cancer screening and treatment?	n/a	Factors that impact men’s supportiveness (e.g. willingness to support their partners at each level of the pathway to care)	Qualitative analysis of survey data: Descriptive statistics - univariate analysis (frequency)

Abbreviations: *CCS* cervical cancer screening, *FGD* focus group discussion, *HPV* Human Papillomavirus, *HR-HPV* high risk HPV, *VIA* visual inspection with acetic acid, *WLWHA* women living with HIV/AIDS

### Participant timeline

A time line of study activities can be found in Table 3.

**Table 3:**
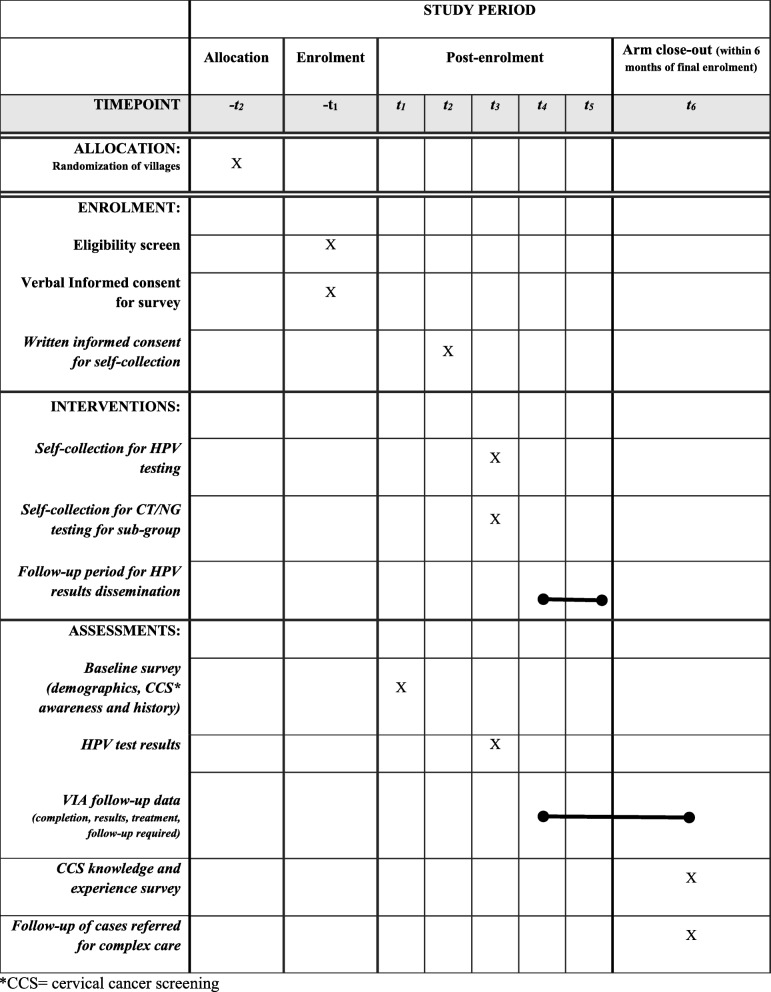
Schedule of study activities for the ASPIRE Mayuge trial

### Sample size

Sample size for the study was restricted to the 31 villages in the region available for randomization. Power was estimated using R statistical software across a range of plausible interclass correlation coefficients (ICCs) and absolute changes in follow-up rate from the baseline of 20% in one study arm. Power calculations assumed 15 clusters per study arm with an average size of 70 women per cluster. Power estimates were repeated using a cluster size of 50 women per village with negligible effect. Using these assumptions, we are adequately powered for a 20–30% absolute change in the outcome rate between the study arms, and up to 10% absolute change if low heterogeneity in clusters is found (see Table 4).

**Table 4:** Power to detect difference in follow-up simulated across a range of ICCs and effect sizes

ICC	10% absolute increase in follow-up	20% absolute increase in follow-up	30% absolute increase in follow-up
0.01	98%	> 99%	> 99%
0.05	71%	> 99%	> 99%
0.10	47%	95%	> 99%
0.20	28%	75%	98%

A sup-group of women from each arm will complete a follow-up survey to assess cervical cancer screening knowledge retention and patient experience within six months of enrollment. Cluster stratified random sampling was used to select a subset of 25 participants from each cluster to total 375 participants from arm 1 and 400 from arm 2 for this survey. The survey sample size was calculated using a fixed number of clusters [23]; an ICC of 0.5; and assuming a mean knowledge score of 8 in arm 1 and 6 in arm 2 with a coefficient of variation of 1 for this score. Based on these assumptions 25 participants per cluster would result in 83% power to detect this target difference.

### Randomization and blinding

The unit of randomization for the trial is the village. Randomization was performed in STATA by BAP, stratified by sub-district (Mayuge and Buwaiswa) using a 1:1 allocation. Stratification was performed to balance the clusters around the two sub-districts that each includes health centres where VIA follow-up is taking place. Blinding of study staff, outcome assessors, or participants is not possible due to the spatial and temporal nature of intervention arms.

### Data collection methods

Primary trial data is captured through a survey of all participants at enrollment; a follow-up survey within 6 months of results; an audit of laboratory result logs; VIA screening logs; and referral and treatment logs at the participating facilities. All enrollment surveys are administered by a trained CHW in Lusoga or English based on the language of preference of the participant. The Core Plus module 1 (CPLUS1) from the Improving Data for Decision Making in Global Cervical Cancer Programs Toolkit-Part 2 (IDCCP) [24] is used to survey eligible women at enrollment. Modifications have been made to the tool to capture participants’ socio-demographic characteristics, preferences on integrated service delivery, and issues related to HIV and other health conditions in collaboration with local partners.

After completion of CCS follow-up and within 6 months of baseline recruitment, an additional subgroup of 775 participants are recruited by a research assistant to complete a knowledge retention and patient-experience survey to better understand inhibitors and facilitators to self-collected CCS behaviour. The CCS knowledge and patient experience survey design was primarily informed by the IDCCP CPLUS1 module [24] and the Organisation for Economic Co-operation and Development’s (OECD) patient-reported experience measures (PREMs) survey tools [25]. Questions regarding knowledge and awareness were taken from Mukama et al.’s [26] survey tool which was previously used to assess CCS knowledge among women in Eastern Uganda. Additional questions were developed to capture motivations for self-collection that are specific to intervention activities.

A supplementary survey is distributed to approximately 300 men in the Mayuge district who attend the community health meetings for arm 2. The men’s survey was designed using the IDCCP CPLUS1 module [24] to capture demographic information, their level of knowledge regarding HPV and cervical cancer and how that informs their attitudes about screening for their partners. It is administered by a CHW and will be conducted in a semi-private location. All responses to the men’s survey remain anonymous and no names are collected.

Cost and person-time data used for cost-effectiveness analysis is collected from the study’s financial records and by using customized survey tools completed at each health centre and through observations of both HPV and VIA screening during each study arm. An audit of the screening logs in each study designated health center is completed at the time of arm 1 recruitment start to estimate a cluster level measure of engagement in the VIA screening program prior to initiation of the study. Focus group discussions with participants who screen positive for HR-HPV and either complete or do not complete follow-up are conducted to evaluate the acceptability of screening and barriers and facilitators to engagement in care.

### Data management and monitoring

A customized data management tool was developed using Research Electronic Data Capture (REDCap) software [27]. Data is entered into the REDCap database by a trained data entry officer and reviewed monthly for completeness. A second reviewer (one of BAP, CA, or JT) randomly selects 10% of the data for independent data quality verification. A data monitoring committee was not established for this trial as no major safety concerns are expected. Furthermore, there are no interim analyses planned and no stopping role. Weekly meetings are held with CHWs to monitor community response and collect information on any adverse or other unintended effects of trial interventions or conduct. Auditing of trial conduct occurs every 3 months by BAP.

### Statistical analysis

#### Primary objective

Statistical analysis will be performed in R Statistical software using the clusterPower package [28] with an intention to treat approach. The primary outcome rate will be computed for each cluster and each study arm and ICCs calculated to assess cluster level variability prior to the primary analysis. The primary effectiveness measure will be compared between arms by estimating a mixed effect logistic regression model with cluster as a random intercept. The model will be adjusted for all known confounders at the individual and cluster level. There is no planned midpoint evaluation as no serious safety concerns are expected.

Apparent prevalence of HPV and STIs will be estimated in the study regions using trial data. Population prevalence of CIN2+ will be estimated based on this apparent prevalence using the reported sensitivity and specificity of the GeneXpert HPV test for this disease status (Table 5).

**Table 5:** Diagnostic test characteristics for moderate and severe cervical lesions

	CIN2+	CIN3
Sensitivity	90.8%(84.7–95.0%)	92.3%(84.8–96.9%)
Specificity	42.6%(38.5–46.9%)	40.0%(36.1–44.0%)

Secondary outcomes compared between arms will be estimated in a similar manner. The effect of each study arm on HPV incidence is estimated using a multi-level logistic regression model as per the primary analysis. The effect of each study arm on CCS knowledge and patient experience will be estimated using a multi-level Poisson model, adjusted for the same individual and cluster level confounders with the addition of HPV status as an individual compounder and with person-time in follow-up as an offset.

Planned secondary analyses include estimating the group by arm interaction of HIV and other STI status on effectiveness of each CCS model. This will be done by adding an interaction term for each comorbidity and arm in the primary model. Temporal trends in HPV incidence and follow-up will be presented graphically.

### Secondary objectives

Cost-effectiveness of the intervention will be estimated using a societal perspective and including all health system and out of pocket costs incurred for women participants in each arm. A decision tree model will be developed based on the patient pathway through care (See Fig. 2) in each study arm and probabilities estimated along the pathway using study data. This decision tree will inform a validated Monte Carlo simulation of HPV data and cervical cancer incidence to compare health outcomes and costs associated with each study arm. Sensitivity analysis will be performed to assess uncertainty around cost estimates, and model inputs such as HR-HPV test sensitivity and specificity and disease progression rates. The primary outcome used will be incremental cost effectiveness ratio and percent reduction in lifetime cervical cancer risk compared between the two study arms.

**Fig. 3:**
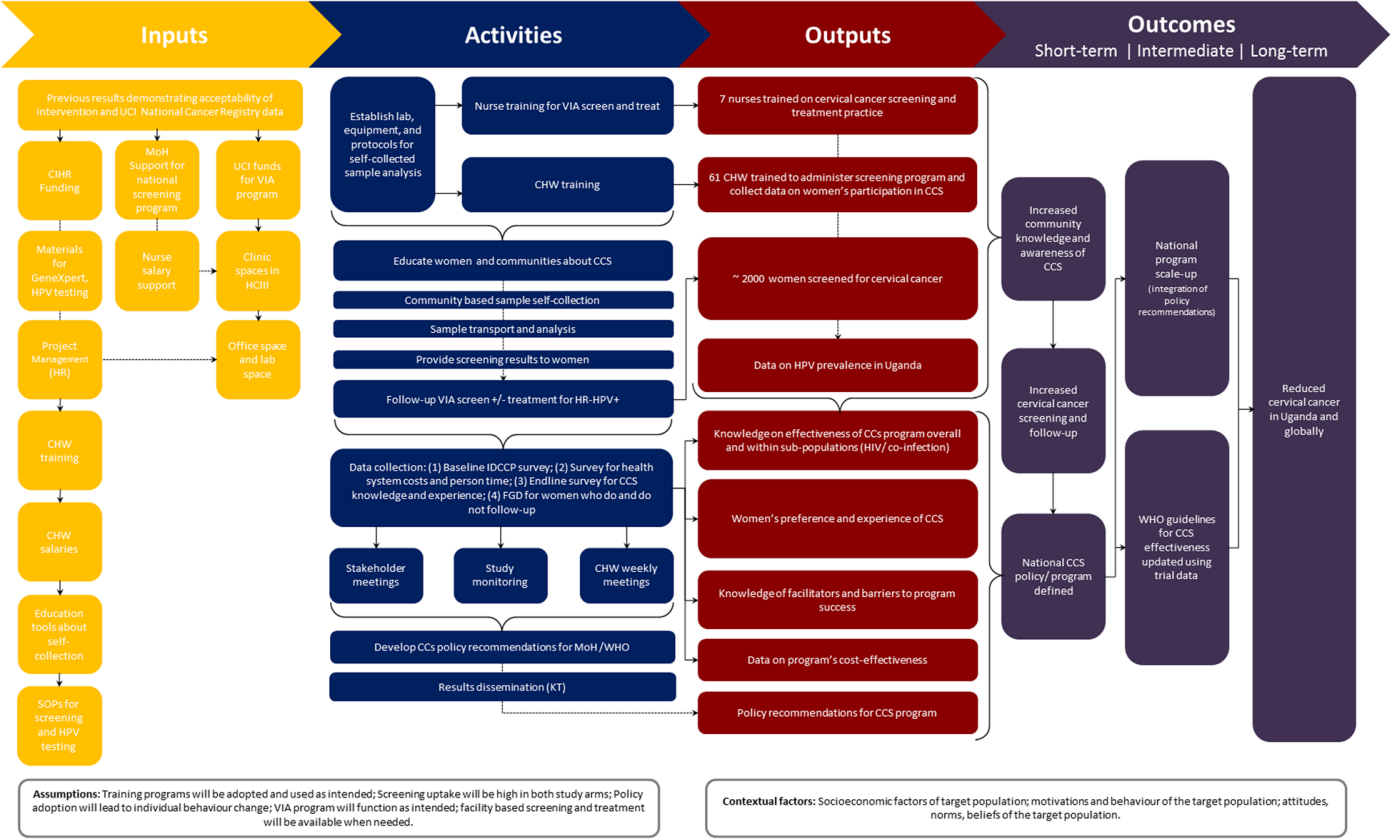
Logic Model

Process evaluation will be performed using a study specific logic model framework to define inputs, activities and outcomes (See Fig. 3.). Process evaluation was informed by the RE-AIM framework [26] and will evaluate reach, acceptability, fidelity and adoption of the screening programs from the participants and health workers perspectives. This analysis will utilize process data collected during implementation of each arm, such as study logs and checklists along with qualitative focus group data and follow-up knowledge and experience survey data.

Qualitative data will be analyzed using InVivo with initial open coding will be done based on the FGD guides to identify themes using a deductive approach.

## Discussion

The ASPIRE Mayuge trial will provide evidence on the best model of delivery for integrated self-collected cervical cancer screening and follow-up in a rural low-resource setting. According to Mayuge District’s Development Plan II, a lack of skilled human resources and infrastructure are the main health system-related challenges faced by the district [19]. To meet the Sustainable Development Goals, the Ugandan Government has prioritized several areas including health infrastructure development and establishing a centre of excellence in cancer treatment and related services [29]. Such investments will help facilitate the Ministry of Health’s strategic plan to expand access to cervical cancer screening by decentralizing screening and VIA to HCIII and providing follow-up care at level IV health centres and hospitals [29].

This approach aligns with the World Health Organization’s efforts towards achieving elimination within the twenty-first century through three key target areas: increased coverage of vaccination against human papillomavirus (HPV); increased screening coverage with an HPV test and appropriate management of women who have screened positive; and reduced mortality from cervical cancer [1]. Ndejjo et al.’s study exploring the uptake of cervical cancer screening among 900 women in Eastern Uganda reveals low screening rates and several challenges to screening [30]. Despite participants having a high level of knowledge about cervical cancer and its risk factors, only 4.8% (*n* = 43) reported ever being screened, 3.6% (*n* = 16) of whom were from Mayuge district [30]. Barriers to screening were characterized by behaviour-related challenges, for example, negative personal perceptions about screening, as well as health system-related challenges such as long wait times, costs, and distance to health facilities.

The major challenges of implementation in real-world global health settings are unsustainable reliance on foreign donors, and the lack of infrastructure, commodities, and human resources [23]. Models proposed in research studies that are entirely funded by foreign donors may not be realistic for scale-up in low resource settings. As a result, models that leverage existing infrastructure and resources, including task shifting to community health workers, need to be explored. Process and outcome evaluation findings from this pragmatic trial will add to the literature on the cost-effectiveness and feasibility of an integrated cervical cancer screening program, and strategies to improve follow-up among those who test positive for HR-HPV in a low-resource rural setting while characterizing the lived experience of participants, health-care providers, and CHWs. Furthermore, men play an important role in health-seeking behaviours, particularly in LMIC [31], and further understanding of the relationship between knowledge and screening could help guide screening protocols. Results from this study will inform the national scale-up of cervical cancer screening in Uganda, aligning with the World Health Organization’s target of achieving cervical cancer elimination through the pillar of increased HPV screening coverage [1].

## Supplementary information

**Additional file 1.** Written Consent Form

**Additional file 2.** Verbal Consent Script: Baseline Survey

**Additional file 3.** Verbal Consent Script: Follow-up Survey

## Data Availability

Data and study materials can be made available upon request and the study protocol and research findings will be published as open access. Both UCI and UBC will retain de-identified copies of the final data set according to our partnership agreement. Final results will be published once the trial is concluded. All publications and data resulting from the trial will be managed by the trial working group (CN, JT, SMF, BAP, AR, NM, CA, HP, JO, LS, GO).

## Abbreviations

ASPIRE: Advances in screening and prevention in reproductive cancers
BCT: Behaviour change technique
BCTTv1: Behavior change technique taxonomy version 1
CCS: Cervical cancer screening
CHW: Community health worker
CIN: Cervical intraepithelial neoplasia
CIN2: Cervical intraepithelial neoplasia grade 2+.
CIN3: Cervical intraepithelial neoplasia grade 3
CPLUS1: Core plus module 1
FGD: Focus group discussion
HCII: Health centre level 2
HCIII: Health centre level 3
HCIV: Health centre level 4
HPV: Human papillomavirus
HR-HPV: High risk subtypes of human papillomavirus
ICC: Interclass correlation coefficients
ICER: Incremental cost-effectiveness ratio
IDCCP: Improving Data for Decision Making in Global Cervical Cancer Programs Toolkit
LEEP: Loop electrosurgical excision procedure
LMIC: Low- and middle-income countries
OECD: Organisation for Economic Co-operation and Development
PREMs: Patient reported experience measures
RE-AIM: Reach, effectiveness, adoption, implementation, maintenance
STI: Sexually transmitted infection
TC: Town council
UCI: Uganda Cancer Institute
VIA: Visual inspection with acetic acid
WLWHA: Women living with HIV/AIDS
