# Supplementary material for: Integrated cervical cancer screening in Mayuge District Uganda (ASPIRE Mayuge): a pragmatic sequential cluster randomized trial protocol
Source: BMC Public Health. 2020 Jan 31;20:142. doi: 10.1186/s12889-020-8216-9 (PMC6995074; doi:10.1186/s12889-020-8216-9)
Supplement: Supplementary file 1 — Additional file 1. Written Consent Form [file 12889_2020_8216_MOESM1_ESM.docx]

Appendix A

Written Consent Form

**CONSENT TO BE SCREENED FOR CERVICAL CANCER and STIs (Door to Door/Community Meeting Screening)**

**Title of the Study:** Integrated Cervical Cancer Screening in Kigandalo Uganda

**Canadian Investigator:** Dr. Gina Ogilvie (University of British Columbia)

**Ugandan Investigator:** Dr. Carolyn Nakisige (Uganda Cancer Institute)

My name is ____________________________. I am a community health worker or research assistant (say only one) in a research study being conducted by researchers at the University of British Columbia in Vancouver, Canada and the Ugandan Cancer Institute in Kampala. We are looking into the best way to provide women with the opportunity to be screened for cervical cancer. In order to gain more information, we request you to participate in cervical cancer screening with self-collection.

Cervical cancer screening will take about 10-15 minutes. You will receive some brief educational information about benefits of cervical cancer screening and its cause (human papilloma virus-HPV), and you will be taught how to self-collect an HPV sample and will be asked to provide two samples to me. This involves you placing a swab like an ear bud in your vagina and then placing it in a container, this should not be painful. One sample will be used to test for HPV and the other sample will be used to test for two other infections that can be spread through sexual contact called gonorrhea and chlamydia. If you do not want to collect and provide the samples you do not have to. If you do provide a sample, the outreach workers will arrange to provide any positive results to you in 2 weeks. If you are found to have HPV, we will inform you of the results, and invite you to attend an assessment at the health centre III. It will involve an examination of your pelvis and your cervix to see if there are any abnormalities. If you require further treatment, this will occur at Kigandalo Health Centre or the Uganda Cancer Institute in Kampala.

We will collect your name and phone number and assign a unique study number as a participant in this study. This number will not include any personal information that could identify you. Only this number will be used on any research-related information collected about you during the course of this study, so that your identity will be kept confidential. Information that contains your identity will remain only with the Principal Investigator and/or designate. The list that matches your name to the unique study number that is used on your research-related information will not be removed or released without your consent.

We are conducting a study to understand how different methods of cervical cancer screening can be integrated into your community. We are offering screening opportunities at door-to-door home visits by community health workers, community health meetings, and local health centres. All eligible women aged 25-49 are encouraged to participate. However, any women who are pregnant, have had a hysterectomy, or cervical cancer are not eligible for this study. Community health workers provide information sheets and explain how to provide a self-collection sample for cervical cancer screening. Once results are available, participants are contacted via phone by nursing staff. If a participant does not provide a mobile phone number, community health workers will attempt to reach participants at their homes. Your community has been randomly chosen to receive (a visit to your door from a community health worker or a community health meeting).

You may or may not personally benefit by participating in this study. By participating in this project you will be able to access HPV and cervical cancer screening and treatment if needed. You will also benefit from being tested and treated for chlamydia and gonorrhea if you have it. You may contribute new information that may benefit people in the future.

The data collected from your participation will be transferred and analyzed in Canada. We will test your specimen for HPV, Chlamydia, and Gonorrhea in a lab.

Your participation in this research is voluntary, and you will not be penalized or impact your access to health services if you refuse to participate or decide to terminate participation at any time. It is important to ensure that you are not pregnant to have the test done. In case you are not sure of your pregnancy status, we shall have you examined and or have a pregnancy test done. There are no known risks to the self-collecting the HPV specimen. It may feel mildly uncomfortable but it should not be painful. The VIA involves a pelvic exam, this can be uncomfortable but should not be painful. If you require treatment of a pre-cancer lesion with cryotherapy (localized freezing of abnormal tissue on the cervix to destroy these abnormalities) this is uncomfortable and will result in vaginal discharge for a few weeks, there is a very small chance you may develop an infection and require some medication to treat this. After a year has passed you will be offered a pelvic examination for follow-up at which time a biopsy will be done. This may hurt for less than a minute and you might have vaginal spotting for a few hours. We will ensure your confidentiality is maintained through this process.

The study staffs, principal investigators, the regulatory staff from Uganda Cancer Institute Ethics Committee (UCIREC) and Uganda National Council for Science & Technology (UNCST) or the UBC Children’s and Women’s Research Ethics Board in Vancouver, Canada can have access to the study documents/data if necessary. This could happen for purpose of study conduct or checking to ensure the research has been done well. All efforts will be made to protect your confidentiality.

In case of any concerns or questions about this study, you may contact the study investigator Dr. Carolyn Nakisige at (number) any time. If you have questions about your rights as a subject for research, you may contact UCIREC at (number). If you feel any distress you can contact the social support worker at the Uganda Cancer Institute (Name&Number).

If you agree to participate, you will be given a signed copy of this document. You will be reimbursed for your travel and time to participate 20,000 shillings.

The research study, as it is written in this consent form has been read and explained to me. I understand the study purpose, benefit and what my involvement in the study means and I voluntarily agree to participate and at any time can change my decision with no penalties.

_____________________________ ______________________ ___________________ Name of Participant Signature/thumbprint Date

**For those that use thumbprint only**:

I attest that participants’ name is ___________________________________________ has voluntarily accepted to take part in this study on this date of ________________________________.

_____________________________ ______________________ ___________________ Name of witness Signature Date

_____________________________ _______________________ ___________________

Name of Research Assistant that Signature Date obtained consent
