# Supplementary material for: Integrated cervical cancer screening in Mayuge District Uganda (ASPIRE Mayuge): a pragmatic sequential cluster randomized trial protocol
Source: BMC Public Health. 2020 Jan 31;20:142. doi: 10.1186/s12889-020-8216-9 (PMC6995074; doi:10.1186/s12889-020-8216-9)
Supplement: Supplementary file 2 — Additional file 2. Verbal Consent Script: Baseline Survey [file 12889_2020_8216_MOESM2_ESM.docx]

Appendix B

Verbal Consent Script: Baseline Survey

Door to door recruitment script Part 1: eligibility

“Hello, my name is _______________________. I am coming to your house today to see if there are any women interested in participating in a study about cervical cancer screening. This study is being run by the Ugandan Cancer Institute and the University of British Columbia, in Canada. Are there any women living here between the ages of 25-49 who I can invite to participate in this study?

If response is yes to part 1 above move on to Part 2: Verbal consent for survey

Door to door recruitment script Part 2: Verbal consent for survey

“The first part of the ASPIRE trial is an anonymous survey. You are invited to complete this survey to help us understand how many women have previously been screened for cervical cancer and how to best design further screening programs. The study is led by Dr Gina Ogilvie at UBC in Canada and Dr Carol Nakisige at the Uganda Cancer Institute. Questions about your information or the survey can be directed to the study leads, their information is on the Consent Form which I will provide you. You do not have to participate. There will be no penalties if you do not want to participate. Your participation is completely anonymous which means it will not contain your name or other personal information. Because this survey collects responses anonymously and cannot link them back to you, it will not be possible to withdraw your consent after you participate. This anonymous data will only be shared with our study team at the University of British Columbia in Canada. The study team will run the survey and analyze the results. Survey data will be stored on a secure network at the Women’s Health Research Institute electronically for 5 years.

Would you be willing to complete the survey?”

If response to recruitment script part 1 is no: thank the person you have been speaking with and move on to the next household.
