# Supplementary material for: Integrated cervical cancer screening in Mayuge District Uganda (ASPIRE Mayuge): a pragmatic sequential cluster randomized trial protocol
Source: BMC Public Health. 2020 Jan 31;20:142. doi: 10.1186/s12889-020-8216-9 (PMC6995074; doi:10.1186/s12889-020-8216-9)
Supplement: Supplementary file 3 — Additional file 3. Verbal Consent Script: Follow-up Survey [file 12889_2020_8216_MOESM3_ESM.docx]

Appendix C

Verbal Consent Script: Follow-up Survey

Follow-up survey script Part 1: Recruitment

“Hello, my name is _______________________. I am coming to your house today to see if the woman living here who participated in the ASPIRE cervical cancer screening study run by the Ugandan Cancer Institute and the University of British Columbia, in Canada is interested in completing a follow-up survey. Is (Participant’s name) available to speak with me?”

If response to Part 1 above is “no”, ask when a good time would be to return, thank the person you have been speaking with and move on to the next household.

If response to Part 1 above is “yes”, move on to Part 2.

Follow-up survey script Part 2: Verbal consent

“You are invited to complete this follow-up survey to help us understand your experience with participating in a cervical cancer screening program using local VHTs and your knowledge of cervical cancer after participating in the study. This will provide us with information on how best to design further screening programs. The study is led by Dr Gina Ogilvie at UBC in Canada and Dr Carol Nakisige at the Uganda Cancer Institute. Questions about your information or the survey can be directed to the study leads. You do not have to participate. There will be no penalties if you do not want to participate. When you participated in screening, we collected your name and phone number, and assigned a unique study number to you as a participant in this study. If you choose to participate in this survey, we will use the same unique study number which does not include any personal information that could identify you. Only this number will be used on any research-related information collected about you during the course of this study, so that your identity will be kept confidential. Information that contains your identity will remain only with the Principal Investigator and/or designate. The list that matches your name to the unique study number that is used on your research-related information will not be removed or released without your consent.

This data will only be shared with our study team at the University of British Columbia in Canada. The study team will run the survey and analyze the results. Survey data will be stored on a secure network at the Women’s Health Research Institute electronically for 5 years.

Would you be willing to complete the survey?”

If response to Part 2: Verbal consent is “no”, thank the person you have been speaking with and move on to the next household.
